# An Ethnic Comparison of Arginine Dimethylation and Cardiometabolic Factors in Healthy Black and White Youth: The ASOS and African-PREDICT Studies

**DOI:** 10.3390/jcm9030844

**Published:** 2020-03-20

**Authors:** Alexander Bollenbach, Aletta E. Schutte, Ruan Kruger, Dimitrios Tsikas

**Affiliations:** 1 Institute of Toxicology, Core Unit Proteomics, Hannover Medical School, 30623 Hannover, Germany; bollenbach.alexander@mh-hannover.de; 2 Hypertension in Africa Research Team (HART), MRC Research Unit for Hypertension and Cardiovascular Disease, North-West University, Potchefstroom 2520, South Africa; a.schutte@unsw.edu.au (A.E.S.); Ruan.Kruger@g.nwu.ac.za (R.K.); 3 School of Public Health and Community Medicine, University of New South Wales, The George Institute for Global Health, Sydney 2052, Australia

**Keywords:** age, atherosclerosis, hypertension, post-translational modification, growth, inflammation

## Abstract

Proteinic arginine dimethylation (PADiMe) is a major post-translational modification. Proteolysis of asymmetric and symmetric PADiMe products releases asymmetric dimethylarginine (ADMA) and symmetric dimethylarginine (SDMA), respectively, two endogenous atherogenic substances. SDMA, ADMA, and its major metabolite dimethylamine (DMA) are eliminated by the kidney. The urinary concentrations of DMA+ADMA, SDMA, and DMA+ADMA+SDMA are useful measures of the whole-body asymmetric and symmetric PADiMe, respectively. Urinary (DMA+ADMA)/SDMA is an index of the asymmetric to symmetric PADiMe balance. In two bi-ethnic studies, the ASOS (39 black boys, 41 white boys) and the African-PREDICT (292 black young men, 281 white young men) studies, we investigated whether ethnicity is a major determinant of PADiMe, and whether PADiMe is associated with blood pressure and ethnicity-dependent growth and inflammatory factors, including HDL. DMA, ADMA, and SDMA were measured in spot urine samples by gas chromatography–mass spectrometry, and their excretion was corrected for creatinine excretion. In black boys, creatinine-corrected DMA, DMA+ADMA, and DMA+ADMA+SDMA concentrations were lower by 11.7%, 9.5%, and 7.6% (all *p* < 0.05), respectively, compared to the white boys, and 3.4%, 2.0%, and 1.8% lower (all *p* < 0.05), respectively, in black compared to white men. (DMA+ADMA)/SDMA did not differ between black boys and black men, but was higher in white boys compared to white men. ADMA did not differ between black and white boys, or between black and white men. Creatinine-corrected SDMA excretion was lower in black boys compared to white boys (by 8%) and to white men (by 3.1%). None of the PADiMe indices were associated with blood pressure in either study. IGF-binding protein 3 correlated inversely with all PADiMe indices in the black men only. Our study showed that asymmetric proteinic arginine dimethylation is higher in white boys than in black boys, and that this difference disappears in adulthood. ADMA metabolism and SDMA excretion were lower in the black subjects compared to the white subjects, suggesting ethnicity-dependent hepatic and renal elimination of ADMA and SDMA in the childhood. The results of our study may have clinical relevance beyond atherosclerosis, such as in growth and inflammation, which have not been sufficiently addressed thus far.

## 1. Introduction

Asymmetric and symmetric dimethylation of L-arginine (Arg) residues of proteins (PADiMe) is an abundant post-translational modification (PTM), which lasts throughout the human lifetime. Arg residues in proteins are methylated on their guanidine (*N*^G^) group by protein-rginine methyltransferases (PRMT; EC 2.1.1.319; EC 2.1.1.320) to form *N*^G^-monomethylarginine (MMA) proteins, which are further *N*^G^-methylated to produce asymmetric *N*^G^,*N*^G^-dimethylarginine (aPADiMe) or symmetric *N*^G^,*N’*^G^-dimethylarginine (sPADiMe) proteins [1,2] (Figure S1). The biological significance of aPADiMe and sPADiMe proteins is poorly understood. There is increasing evidence that proteins, including those in transporters and channels, are methylated on particular Arg residues and that PADiMe alters the biological activity of native proteins [1,2,3,4,5,6].

Proteolysis of *N*^G^-monomethylarginine, aPADiMe, and sPADiMe proteins releases the free amino acids MMA, ADMA, and SDMA, respectively. MMA and ADMA are hydrolyzed mainly by hepatic and renal dimethylarginine dimethylaminohydrolase (DDAH; EC 3.5.3.18) to form monomethyl amine and dimethyl amine (DMA), respectively (Figure S1). SDMA is not metabolized by DDAH. MMA, ADMA, SDMA, and DMA circulate in blood at nM-to-µM concentrations and are excreted in the urine [7,8]. In adults, about 90% of the ADMA produced daily is estimated to be hydrolyzed to DMA, which is excreted in the urine, with the remaining 10% of ADMA being excreted unchanged in the urine [9]. The concentrations of MMA in blood and urine are considerably lower than those of ADMA and SDMA. In adults, SDMA and ADMA are excreted in comparable µM concentrations in urine [7,8]. DMA is the major ADMA metabolite and is found at concentrations in the lower µM range in the blood, and in the upper µM range in urine. In adults, the creatinine-adjusted excretion rates are about 4 µM/mM for both ADMA and SDMA, and 30 µM/mM for DMA [10]. To our knowledge, the excretion of DMA has not been assessed in bi- or multiethnic studies to date.

The free amino acids MMA, ADMA, and SDMA have received particular attention because they are endogenous inhibitors of nitric oxide synthase (NOS; EC 1.14.13.39) activity. They act via distinctly different mechanisms and with different potency, with the approximate order being MMA>ADMA>>SDMA for neuronal NOS (nNOS) activity [11,12]. NOS isoforms include the constitutive nNOS and endothelial NOS (eNOS), and the inducible NOS (iNOS). NOS converts Arg to nitric oxide (NO) and L-citrulline [11,12]. NO is one of the most potent endogenous vasodilators and regulators of blood pressure (BP) [13]. In adults, elevated circulating ADMA concentrations are associated with hypertension [9]. ADMA and SDMA are considered cardiovascular risk factors and have emerged as predictors of cardiovascular events and death in a range of diseases in adults [14]. The ADMA-related cardiovascular risk is generally assumed to be deeply rooted in the ability of ADMA to inhibit eNOS-catalyzed synthesis of NO in the endothelium [11,12,15]. A diminished NO bioavailability resulting from inhibition of eNOS activity by elevated MMA, ADMA, and SDMA concentrations may lead to hypertension in adults. However, healthy newborns and children have about 2-fold higher circulating concentrations of ADMA compared to adults [16,17], without any signs of endothelial dysfunction, hypertension, or cardiovascular or renal disease. The plasma concentration of ADMA decreases constantly with increasing age until adulthood, from about 1 µM to 0.4 µM with a mean rate of about 17 nM per year [16]. In adults older than 25 years, the blood concentration of ADMA increases with age, but at a much lower rate [18] compared to its decrease rate from birth to adulthood [16]. 

The cardiovascular risk of ADMA is generally attributed to its inhibitory action on eNOS activity. However, the inhibitory potency of ADMA towards recombinant human eNOS is very low (IC_50_, 12 µM) and its concentration in blood and tissue is too low to enable inhibition of eNOS activity to an extent that can raise BP [11,19]. SDMA is generally assumed not to inhibit eNOS activity. We hypothesized that not the inhibitory action of the free acids of ADMA and SDMA on eNOS activity, but other not yet known, NO-independent activities of ADMA, SDMA, and their peptidic/proteinic precursors are primarily responsible for the cardiovascular risk associated with high circulating ADMA and SDMA concentrations [11,12]. For example, plasma ADMA and SDMA concentrations were found to be higher both in hypothyroidism and hyperthyroidism than in healthy subjects [20]. Circulating ADMA and SDMA, but not circulating Arg and its methylene homolog L-homoarginine (hArg), both substrates of NOS, were found to be positively associated with low thyrotropin, free T3, and free T4 in the general population, suggesting that the atherogenic properties of ADMA and SDMA may be partially mediated by thyroid function [21].

Age, sex, disease, and other factors such as smoking are known to influence synthesis, metabolism, and elimination of free ADMA and SDMA. It is widely known that in South Africa hypertension is a public health problem, with ethnicity being a major determinant of hypertension (see references below). The µM concentrations of ADMA, DMA and SDMA in human urine suggest that PADiMe is an abundant PTM, with aPADiMe being more abundant than sPADiMe. However, the determining factors and significance of this particular PTM are poorly understood. Previous studies have suggested that ethnicity may also contribute to the differences found for circulating and excretory ADMA in multi-ethnic populations [22,23,24,25,26,27,28]. 

To the best of our knowledge, no data are available for different ethnicities for whole-body aPADiMe and sPADiMe, for their mutual relationship, or for their associations with other ethnicity-dependent determining factors, such as growth and inflammatory factors. The aim of the present study was to investigate potential associations of ethnicity, mainly represented by various demographic and anthropometric characteristics, with whole-body PADiMe in two bi-ethnic populations from South Africa. We applied an entirely non-invasive, previously described and evaluated approach [29] to measure the concentration of urinary ADMA, SDMA, and DMA and to determine four indices of whole-body PADiMe (Figure S1) in two studies: (1) in healthy black and white boys from the Arterial Stiffness in Offspring Study (ASOS) [30,31]; and (2) in healthy black and white young men from the African Prospective Study on the Early Detection and Identification of Cardiovascular Disease and Hypertension (African-PREDICT) [32]. In the African-PREDICT study, urine samples were also collected from women, and the PADiMe was also assessed in this group for comparison. As spot urine samples were collected in both studies, the concentrations of urinary ADMA, SDMA, and DMA were corrected for creatinine excretion. The PADiMe indices (PADiMeX) estimated in the present study were defined as follows (Figure S1).

1. [DMA] + [ADMA]      asymmetric PADiMe (aPADiMeX)
2. [SDMA]           symmetric PADiMe (sPADiMeX)
3. [DMA] + [ADMA] + [SDMA] total PADiMe (toPADiMeX)
4. ([DMA] + [ADMA])/[SDMA] asymmetric/symmetric PADiMe (a/sPADiMeX)

In these terms, [DMA], [ADMA], and [SDMA] are the concentrations (in µM) or creatinine-corrected excretion rates of DMA, ADMA, and SDMA (in µM/mM) in the urine samples, respectively. 

On the basis of these indices, we tested whether whole-body PADiMe was associated with BP and other potentially ethnicity-dependent growth and inflammatory clinical factors including insulin-like growth factor-1 (IGF1), interleukin-6 (IL-6), tumor necrosis factor alpha (TNF-α), and high-density lipoprotein (HDL) in the investigated studies.

## 2. Experimental Section

### 2.1. Investigated Studies

We included complete datasets for urinary ADMA, DMA, SDMA, and creatinine of black (*n* = 39) and white (*n* = 41) healthy boys (aged 6–8 years) from the ASOS study, and of black (*n* = 292) and white (*n* = 281) healthy men (aged 19–31 years) and black (*n* = 309) and white (*n* = 312) healthy women (aged 19–31 years) from the African-PREDICT study. 

Children aged younger than 6 years or older than 8 years were excluded from the ASOS study [30]. Girls were also excluded because of potential hormonal influences of unknown pubescence onset. Further exclusion criteria were obese children whose body mass index z-scores were over the 95th percentile, as indicated by the World Health Organisation (WHO), children using any chronic medication, or with self-reported type 1 diabetes mellitus, renal disease, or cancer. 

The study population and the protocol of the African-PREDICT study have been described in detail elsewhere [32]. Briefly, exclusion criteria were participants with screening office BP > 140/90 mmHg, or with any self-reported diseases or risk factors that might influence cardiovascular health, internal ear temperature > 37.5 °C, human immunodeficiency virus (HIV), diabetes mellitus, liver disease, cancer, tuberculosis, or renal disease, or the use of chronic medication. Pregnant and lactating women were excluded due to known influences of hormones on cardiovascular health.

Participants were fully informed about the objectives of the studies and consent and assent forms were duly signed. Both studies were conducted in line with the ethical principles of the Declaration of Helsinki [33] and were approved by the Health Research Ethics Committee of the North-West University (NWU-00007-15-A1 for the ASOS study; NWU-00001-12-A1 for the African-PREDICT study). The African-PREDICT study is also registered at ClinicalTrials.gov (identifier: NCT03292094).

### 2.2. Anthropometric Measures 

All anthropometric procedures were performed according to specific guidelines set out by the International Society for the Advancement of Kinanthropometry (ISAK) [32,34]. Body mass index (BMI) (kg/m^2^) of each participant was calculated (SECA portable 213 stadiometer; SECA 813 electronic scale; Hamburg, Germany). BMI z-scores were used for the assessment of body composition in children. Thresholds derived from a child growth reference were used to classify the BMI z-scores of children according to their age and sex [35].

### 2.3. Cardiovascular Measures 

In the ASOS study, brachial BP was measured in triplicate on the upper left arm at heart level with a validated pediatric Omron HEM-759-E (750IT) device (Omron Healthcare, Tokyo, Japan) [36]. With the use of a Dinamap^®^ ProCare 100 Vital Signs Monitor, brachial BP of the African-PREDICT participants was measured on the left arm, and thereafter on the right arm in duplicate followed by a repeated measure on the left upper arm (GE Medical Systems, Milwaukee, USA). In this study, the left BP measurement was used in the analyses. Systolic blood pressure (SBP) and diastolic blood pressure (DBP) were captured from each measurement. The mean arterial pressure (MAP) was subsequently calculated from brachial BP recordings. MAP was calculated using the formula: MAP = DBP/(0.4 × pulse pressure) [37].

### 2.4. Urine Collection and Measurement of Urinary ADMA, DMA, SDMA, and Creatinine

A first voided mid-stream urine sample was acquired from each ASOS participant in the privacy of their own home. Participants were provided with sealable cups and containers to collect first urine samples on the day of participation. The African-PREDICT participants were required to provide an early-morning spot urine sample at the Hypertension Clinic of the North-West University. Urine samples were stored frozen and aliquoted at −80 °C and sent to the Hannover Medical School (Hannover, Germany) in dry ice, where they were stored at −20 °C until analysis. After thawing, urinary metabolites were measured in 10 µL aliquots of urine samples by using previously reported and fully validated methods based on gas chromatography–mass spectrometry (GC-MS) for DMA [10], ADMA [38], SDMA [39], and creatinine [40]. Urinary concentrations of DMA, ADMA, and SDMA (in µM) were divided by the respective urinary concentrations of creatinine (in mM), and the creatinine-corrected excretion rates are reported in µM/mM.

### 2.5. Clinical Chemistry Measures

Urinary electrolytes and all blood biomarkers were measured using standard clinical chemistry assays. Urinary concentrations of electrolytes (in mM) were divided by the respective urinary concentrations of creatinine (in mM), and their creatinine-corrected excretion rates are reported in mM/mM.

### 2.6. Statistical Analysis

Statistical analyses were performed via IBM SPSS Statistics 19 and GraphPad Prism 7 (GraphPad Prism Software Inc. San Diego, California, USA). Distribution of variables was tested by D’Agostino and Pearson omnibus K2 test. Normally distributed parameters are presented as mean ± standard deviation (SD). Non-normally distributed parameters are presented as median with interquartile range (IQR, 25th–75th percentile). Normally and non-normally distributed parameters in subgroups are presented as median with IQR. Correlations between variables were assessed by Pearson (parametric) or Spearman (non-parametric). Statistical significance was assumed for *p* values < 0.05. One-way ANOVA with Tukey correction was performed for comparison between groups.

## 3. Results

The demographic and anthropometric characteristics of the participants are summarized in Table 1 for the ASOS study, in Table 2 for the African-PREDICT study, and in Table 3 for the comparison of both studies. 

### 3.1. The ASOS study

Most of the anthropometric characteristics of the white and black boys were similar. White boys had higher neck circumference (3.6%) and waist-to-height ratio (7.3%) (Table 1). DBP (by 8.7%) and clinic MAP (by 6%) were higher in the black compared to the white boys (Table 1). There were no differences regarding urinary clinical chemistry measures between white and black children. Urinary creatinine concentration did not differ between white and black boys (Table 1). White boys had higher (by 11.7%) creatinine-corrected excretion rates of DMA compared to black boys. This difference is likely to have contributed to the statistical significance with respect to aPADiMeX (by 9.5%) and toPADiMeX (by 7.6%). The difference for SDMA (by 8%) between white and black boys was borderline significant (*p* = 0.055). The creatinine-corrected excretion of ADMA did not differ between black and white boys. No differences were found for a/sPADiMeX or the ADMA/SDMA molar ratio.

### 3.2. The African-PREDICT Study

The ages of the white and black men of the African-PREDICT study were similar (Table 2). White men had higher body weight, stature, BMI, circumference of waist, neck, and hip, and waist-hip ratio values by 5% to 27% (all *p* < 0.0001) than black men. The urinary creatinine concentration was higher (by 10.9%) in white men (Table 2). White men also had higher (by 3.4%) creatinine-corrected excretion rates of DMA compared to black men. The creatinine-corrected excretion of SDMA (by 3.1%), but not of ADMA, was higher in white compared to black men. White men had also higher aPADiMeX and toPADiMeX compared to black men, although these differences were rather small (each by about 2%). No differences were found for a/sPADiMeX between white and black men. However, the median ADMA/SDMA molar ratio was higher (by 8%) in the black men compared to the white men (*p* = 0.0001) (Table 2).

### 3.3. Comparison of the Arg-Dimethylation Indices between Boys and Men (Table 3)

Urinary creatinine concentrations of the white boys were higher (by 13.2%) than of the white men, with borderline significance (*p* = 0.053), while the difference between black boys and men was not significant (*p* = 0.122). The urinary creatinine concentration was lower in the men compared to the boys by a mean factor of 1.15 in the white subjects, and by a mean factor of 1.28 in the black subjects. In white subjects, all PADiMe indices were higher in the boys compared to the men. In the black subjects, all PADiMe indices, with the exception of a/sPADiMeX (*p* = 0.258), were significantly higher in the boys compared to the men. On average, the creatinine-corrected excretion rates were lower in the men compared to the boys by mean factors of 1.68 and 1.71 for ADMA, 1.46 and 1.28 for DMA, and 1.36 and 1.29 for SDMA in white and black subjects, respectively. 

### 3.4. Correlations in the ASOS and African-PREDICT Studies

The results of the correlations of the urinary concentrations of DMA, ADMA, SDMA, and their sum (i.e., toPADiMeX) with creatinine, themselves, blood pressure, and urinary and other parameters among black and white subjects, as well as among the combined groups are presented in Tables S1 and S2 (see Supplementary Material). In both the ASOS and African-PREDICT studies, we found close inter-correlations between all protein Arg-dimethylation indices and creatinine.

In the ASOS study, DMA, ADMA, SDMA, and toPADiMeX correlated moderately with the urinary concentrations of chloride, potassium, and sodium only in the black boys (Table S1). We found no statistically significant correlations of DMA, ADMA, SDMA, and toPADiMeX with age, BMI, weight, waist circumference, neck circumference, hip circumference, or waist-to-height ratio in the white and black boys (Table S1). None of the PADiMe indices correlated with SBP, DBP, or MAP in the black and white boys (Table S1).

In the African-PREDICT study, we found mostly weak positive correlations of DMA, ADMA, SDMA, and toPADiMeX, with the majority of the anthropometric characteristics only in the black men (Table S2). DMA, ADMA, SDMA, and toPADiMeX correlated positively with blood glucose in the black men (Table S2), while ADMA correlated inversely with blood glucose in the white men. Blood IGFBP-3 correlated inversely with DMA, ADMA, SDMA, and toPADiMeX in the black men, while these parameters correlated positively with blood IL-6 (Table S2). 

### 3.5. Arg Dimethylation Indices in the Women of the African-PREDICT Study

In the African-PREDICT study, we collected spot urine samples from age-matched black women (*n* = 309) and white women (*n* = 312) and measured urinary creatinine, DMA, ADMA, and SDMA. The results of these analyses and the Arg-dimethylation indices are summarized in Table 4. DMA, SDMA, (DMA+ADMA+SDMA), and SDMA/ADMA were higher by approximately 7%, 7%, 6%, and 11% in the white compared to the black women, respectively.

### 3.6. Comparison of the Arg-Dimethylation Indices between Men and Women in the African-PREDICT Study

The results from the comparison of the Arg dimethylation indices between men and women in the African-PREDICT study are summarized in Table S3. White women had lower BMI values than white men, while black women had higher BMI values than black men. White women had higher urinary creatinine concentrations than white men, while black women had lower urinary creatinine concentrations than black men. Creatinine-corrected excretion rates of DMA (by 12% and 8%), ADMA (by 20% and 21%) and SDMA (by 16% and 12%) were higher in the white and black women compared to the white and black men, respectively. White women excreted more DMA (by 7%) and SDMA (by 7%) in their urine than black women. In contrast, black women excreted more ADMA (by 4%) in their urine than white women. Total Arg dimethylation (DMA+ADMA+SDMA) was higher in the white (by 14%) and black (by 10%) women compared to white and black men, respectively. There was no preference for asymmetric or symmetric Arg dimethylation (i.e., a/sPADiMeX) in the groups of women (*p* = 0.422).

## 4. Discussion

### 4.1. Arg Dimethylation and Blood Pressure

NO is a potent endogenous vasodilator and regulator of BP produced from L-arginine by the catalytic action of NOS [13]. The free amino acids MMA, ADMA, and SDMA are NOS inhibitors. This action is generally believed to be responsible for their atherogenic and BP-enhancing effects. Given the weak inhibitory potency of ADMA and SDMA towards eNOS activity [11,19,41], we hypothesized that not free ADMA and SDMA, but rather their proteinic precursors may be responsible for the cardiovascular risk, which is associated with high blood circulating and low urinary concentrations of ADMA and SDMA in the general population. In South Africa, hypertension is a major public health problem. The Birth to Twenty study (*n* = 3273; 78.5% black) revealed a high prevalence of hypertension which tracked from early childhood (5 years) into late adolescence (18 years) [42], indicating ethnicity as a major determinant of hypertension. Previous multi-ethnic studies have referred almost exclusively to free circulating ADMA [22,23,25,26,27,28,29]. In previous bi-ethnic studies, serum ADMA and SDMA concentrations were found not to differ between black and white men [43]. In the SABPA (Sympathetic Activity and Ambulatory Blood Pressure in Africans) study, black men and women had higher BP and albumin-to-creatinine ratios, higher plasma concentrations of the NOS substrate Arg, and even lower plasma concentrations of ADMA and SDMA, the NOS inhibitors, compared to white men [28,44]. 

The bi-ethnic studies ASOS and African-PREDICT were originally conducted to investigate the link of urinary metabolites with premature arterial stiffness and the early detection and identification of cardiovascular disease and hypertension development in black and white populations from South Africa [31,32]. The ASOS study included black and white boys only, whereas the African-PREDICT included young black and white men and women. The aims of the present study were to investigate whether ethnicity is a major determinant of whole-body proteinic asymmetric and symmetric Arg dimethylation (PADiMe), and whether PADiMe is associated with BP and ethnicity-dependent anthropometric characteristics and clinical measures including growth and inflammatory factors. Previously, we demonstrated the utility of the PADiMe approach and its independency of fat-rich protein consumption in healthy overweight men [29]. In the present study, we applied this approach to the ASOS and African-PREDICT studies. In previously collected spot urine samples from these studies, we measured the urinary excretion of ADMA, DMA, SDMA, and creatinine, calculated PADiMe indices, and used them as measures of whole-body PADiMe.

In both the children from the ASOS study and the adults from African-PREDICT study, the white subjects presented with higher mean creatinine-corrected urinary excretion rates of DMA, DMA+ADMA (i.e., total asymmetric Arg dimethylation) and DMA+ADMA+SDMA (total asymmetric + symmetric Arg dimethylation). The creatinine-corrected urinary excretion rate of SDMA was higher in the white boys and white men. However, these changes did not influence the asymmetric-to-symmetric Arg dimethylation balance a/sPADiMeX, i.e., (DMA+ADMA)/SDMA. None of the PADiMe indices were found to be associated with BP in the ASOS and African-PREDICT studies, which included healthy participants. The findings of the present study support the observations of the lower circulating ADMA and SDMA concentrations in black subjects measured in the SABPA study [44]. Low urinary excretion of ADMA is a cardiovascular risk factor in white adults [45,46]. The close comparability of urinary ADMA excretion in the young white and black men of the African-PREDICT study suggests that the black population is not exposed to a higher ADMA-associated cardiovascular risk than the white population, but, again, it should be taken into account that the study included healthy screening normotensives. 

The black boys of the ASOS study were found to have increased arterial stiffness in all sections of the arterial tree, along with higher DBP (by 8.7%; Table 1), carotid intima-media thickness, and advanced glycation end-products [30]. Interestingly, in the young men of the African-PREDICT there was no difference in DBP, while the white young men had higher SBP (by 1.6%; Table 1). 

ADMA and SDMA, and possibly their proteinic precursors, may exert additional not yet recognized effects. For example, the atherogenic effects of ADMA and SDMA are considered to be partially mediated by alteration of the thyroid function [20,21]. Such presumably NO-independent effects may contribute to the adverse cardiovascular profile found in black populations. In the SABPA study, plasma ADMA and SDMA concentrations were found to be independently associated with carotid intima-media thickness in the black men [47]. In white stroke patients, we found that plasma ADMA concentration correlated with aortic intima-media thickness, but not with aortic distensibility, suggesting different underlying mechanisms for aortic remodeling [48].

Thus far, the highest PADiMe indices have been measured in preterm neonates [17], followed by the boys of the ASOS study and the young men of the African-PREDICT studies reported in this work. The extent of whole-body PADiMe is highest at birth and decreases with age, but without appreciable changes of the asymmetric-to-symmetric PADiMe balance during the lifetime. This is an important assessment and argues for a potential role of growth factors in protein Arg dimethylation.

### 4.2. Arg Dimethylation and Growth and Inflammatory Factors

The concentrations of plasma IGFBP-3, but not of IGF-1, were higher in the white compared to the black men of the African-PREDICT study. In this population, circulating IGFBP-3 correlated inversely with the excretion rates of ADMA, DMA, and SDMA in the black men of the African-PREDICT study only. The IGF-1-to-IGFBP-3 molar ratio, a surrogate of free IGF-1, was lower in the white men compared to black men and correlated positively with the excretion rate of ADMA and SDMA, but not of DMA in the whole cohort of men. This observation may suggest that the PRMT activity, i.e., the synthesis of ADMA and SDMA, but not the DDAH activity, i.e., the metabolism of ADMA to DMA, is associated with the IGF-1-to-IGFBP-3 molar ratio. A recent study showed that activation of the IGF-1R by IGF-1 activates PRMT1 which, in turn, activates ERα via Arg methylation in a ligand-independent way [49]. Subsequently, activated ERα triggers the AKT/ERK-signal pathway, ultimately leading to growth stimulation. It has been reported that ERα, being methylated on Arg residues, undergoes degradation by the proteasome, thus contributing to free ADMA [50]. The lower IGFBP-3 concentrations found in the black men of the African-PREDICT study could attenuate the activity of IGF-1R, thus reducing PRMT1-catalyzed ADMA biosynthesis. 

In healthy overweight men, creatinine-corrected urinary excretion rates of ADMA, DMA, and SDMA were found to correlate positively with circulating TNF-α and IL-6 [29]. In the African-PREDICT study, circulating IL-6 correlated positively with the excretion rate of DMA in the black men, and of ADMA in the black and the white men. This parallelism may suggest that asymmetric Arg dimethylation is favored by inflammation in both black and white populations.

High-density lipoprotein (HDL) is generally considered to be anti-atherogenic and is known to prevent endothelial dysfunction [51]. Free SDMA has been reported to cause the transformation of physiological HDL to an abnormal atherogenic HDL, which reduces endothelial NO availability by enhancing endothelial superoxide production via activation of the toll-like receptor-2 (TLR-2) [52]. However, the transformation of physiological HDL to an abnormal atherogenic HDL was not further characterized in that study [52]. In the African-PREDICT study, black men differed in their lipid profiles, including the total cholesterol-to-HDL and LDL-to-HDL molar ratios, which may translate into a higher cardiovascular risk [53,54]. However, in the African-PREDICT study, urinary ADMA, but not urinary SDMA, correlated inversely with plasma HDL and LDL in the white men only. It remains to be investigated whether HDL is transformed differently to an abnormal atherogenic HDL in black and white people.

### 4.3. Potential Limitations of the Study

Our studies were well planned and executed under strict conditions. The populations included participants from the northwest province of South Africa and are not representative of the population as a whole. Because of the cross-sectional design, we were unable to elucidate underlying mechanisms and causal relationships. Future studies on proteinic Arg dimethylation should include girls when these associations are explored in children. Future studies should also consider diet in order to minimize artefactual contributions of dietary DMA to ADMA-derived DMA [29]. Of particular importance is abstinence from fish and seafood, which are rich in DMA [55]. We have shown that intake of canned fish resulted in several-fold increases in urinary DMA concentrations and excretion rates [10]. In the present studies, the ranges of urinary DMA values were narrow, suggesting no artefactual contribution of diet to DMA. Met is an essential amino acid and the precursor of *S*-adenosyl-methionine (SAM), the common cofactor of PRMT and other methyltransferases. Arg is a semi-essential amino acid and the common precursor of all PADiMe indices. To date, there is no convincing evidence that diet or supplementary Met and Arg reasonably contributes to PADiMe, indices including circulating and excretory ADMA and SDMA [56,57,58,59,60]. In both studies, we performed analyses of urine samples collected by spontaneous micturition, and the urinary excretion of DMA, ADMA, and SDMA therefore had to be corrected for the creatinine excretion. The diurnal variability of the urinary excretion of DMA, ADMA, and SDMA is another factor that may have contributed to the differences found in our studies. However, the diurnal variability of circulating and urinary DMA, ADMA, and SDMA are relatively low [61], and is unlikely to have affected the outcome of our studies. Due to the relatively narrow age ranges (6 to 8 years in the ASOS study and 19 to 31 years in the African-PREDICT study), an age-dependency of DMA, ADMA, and SDMA is unlikely to have affected the results from either study.

## 5. Conclusions and Perspectives

The concentrations of ADMA, SDMA, and DMA in the urine samples analyzed in the present study correlated closely with the concentration of creatinine in urine. Creatinine-corrected excretion rates allow for the non-invasive and reliable estimation of whole-body proteinic Arg dimethylation (PADiMe), even if urine is collected by spontaneous micturition. PADiMe differs between black and white children, decreases with age, and became indistinguishable in these South African bi-ethnic populations. Based on the currently available information, the largest changes in PADiMe occur in the first years of life. Growth factors are likely to be major determinants of PADiMe in the childhood and inflammatory factors in adulthood. Proteinic Arg dimethylation, as measured by us in both South African bi-ethnic populations, was not associated with BP in black and white boys and young men. 

Red blood cells may modulate vascular function by various mechanisms, including release of vasodilatory molecules such as NO and ATP [62]. Conditions where erythrocytes adhere to the endothelium can result in hypertension and vasoconstriction. Major erythrocytic membrane proteins, which include spectrin-α, spectrin-β, ankyrin-1, and protein band 4.1, are Arg-dimethylated proteins [63]. Altered erythrocyte membrane protein composition has been shown to mirror the pleiotropic effects of hypertension susceptibility genes and disease pathogenesis [64]. The role of these Arg-dimethylated erythrocytic proteins in vascular function modulation is unknown. Both genetic assays and biochemical measurements of Arg-dimethylated proteins in the erythrocytic membrane are required to elucidate whether Arg dimethylation is an ethnicity-dependent contributor to the early development of raised BP in black and white populations from South Africa, or in other populations.

## Figures and Tables

**Table 1:** Anthropometric characteristics, urinary clinical chemistry parameters, and urinary creatinine and creatinine-corrected indices of L-arginine dimethylation in the boys of the ASOS study.

Parameter	White (*n* = 41)	Black (*n* = 39)	*p*-Value	Mean Δ(white−black) (%)
Characteristics				
Age (years)	7.68 ± 0.97	7.84 ± 0.72	0.426	
BMI (kg/m^2^)	16 (15–17)	16 (15–17)	0.238	
Weight (kg)	23.3 (22.1–28.5)	25.1 (23.0–28.6)	0.303	
Height (cm)	127 (120–132)	125 (122–129)	0.772	
Waist circumference (cm)	56 (54–61)	56 (53–60)	0.432	
Neck circumference (cm)	27.5 ± 1.73	26.5 ± 1.56	**0.004**	**+3.6**
Waist to height ratio	0.86 ± 0.04	0.83 ± 0.03	**0.0001**	**+3.5**
SBP (mmHg)	102 ± 7	105 ± 11	0.160	
DBP (mmHg)	63 ± 8	69 ± 9	**0.001**	**−8.7**
MAP (mmHg)	78.7 ± 6.61	83.7 ± 9.21	**0.006**	**−6.0**
**Clinical chemistry (urine)**				
Chloride (mM/mM)	8.81 (6.55–11.7)	10.8 (6.04–19.9)	0.299	
Potassium (mM/mM)	0.59 (0.41–0.90)	0.54 (0.45–0.90)	0.684	
Sodium (mM/mM)	1.89 (1.40–2.81)	2.03 (1.44–3.14)	0.509	
**Urinary metabolites**				
Creatinine (mM)	15.9 (12.8–18.7)	15.3 (10.1–21.4)	0.496	
DMA (µM/mM)	38.4 (33.8–46.8)	33.9 (29.0–38.8)	**0.017**	**+11.7**
ADMA (µM/mM)	5.52 ± 0.94	5.72 ± 1.26	0.442	
SDMA (µM/mM)	4.89 ± 0.89	4.50 ± 0.80	0.055	
DMA+ADMA+SDMA (µM/mM)	48.5 (43.1–58.5)	44.8 (39.3–49.9)	**0.026**	**+7.6**
DMA+ADMA (µM/mM)	44 (39.0–52.7)	39.8 (35.2–45.3)	**0.025**	**+9.5**
(DMA+ADMA)/SDMA	8.97 (7.77–10.1)	8.66 (7.70–9.87)	0.592	
ADMA/SDMA	1.14 (1.00–1.32)	1.21 (1.10–1.44)	0.105	

Data are presented as mean ± SD, percentage or median [IQR], and mean percentage difference Δ between white and black subjects. Statistical analysis was performed using Student’s *t* test or Mann–Whitney *U* test as appropriate. Bold indicates statistical significance (*p* < 0.05).

**Table 2:** Anthropometric characteristics, plasma clinical laboratory parameters, and urinary creatinine and creatinine-corrected indices of L-arginine dimethylation in the men of the African-PREDICT study.

Parameter	White (*n* = 281)	Black (*n* = 292)	*p*-Value	Mean Δ(white−black) (%)
Characteristics				
Age (years)	25 (22–27)	24 (22–27)	0.089	
BMI (kg/m^2^)	26.2 (23.4–29.1)	21.4 (19.4–24.2)	**0.0001**	**+18.3**
Weight (kg)	84.2 (75.1–94.2)	61.6 (55.1–72.2)	**0.0001**	**+26.8**
Height (cm)	179.1 ± 6.22	170 ± 6.69	**0.0001**	**+5.1**
Waist circumference (cm)	87.5 (81.1–94.5)	74.9 (69.9–81.2)	**0.0001**	**+14.4**
Neck circumference (cm)	39.0 (37.1–40.8)	35.1 (34.0–36.7)	**0.0001**	**+10.0**
Hip circumference (cm)	104 (97.7–110)	92.3 (86.5–99.5)	**0.0001**	**+11.3**
Waist to hip ratio	0.85 (0.81–0.89)	0.81 (0.77–0.86)	**0.0001**	**+4.7**
Ear temperature (°C)	36.5 (36.2–36.7)	36.4 (36.1–36.7)	**0.003**	**+0.3**
SBP (mmHg)	122 (116–129)	120 (113–130)	**0.022**	**+1.6**
DBP (mmHg)	80 (75–86)	81 (75–86)	0.534	
Pulse pressure (mmHg)	44.0 (39.0–49.3)	41.3 (35.6–46.5)	**0.0001**	**+6.8**
MAP (mmHg)	97.7 (93.0–103.3)	96.8 (91.8–102)	0.324	
Smokers, *n* (%)	80 (28.5 %)	125 (42.8 %)	**0.0001**	**−33.4**
Alcohol drinkers, *n* (%)	165 (58.9 %)	181 (62.0 %)	0.517	
**Clinical chemistry (plasma)**				
Total cholesterol (mM)	4.03 (2.79–4.94)	3.16 (2.62–3.86)	**0.0001**	**+21.6**
LDL (mM)	2.63 (1.83–3.52)	1.93 (1.47–2.52)	**0.0001**	**+26.6**
HDL (mM)	0.93 (0.71–1.16)	1.04 (0.81–1.33)	**0.0001**	**−10.6**
Total cholesterol/HDL	4.07 (3.31–4.98)	2.90 (2.43–3.57)	**0.0001**	**+28.7**
LDL/HDL	2.84 (2.10–3.64)	1.82 (1.39–2.42)	**0.0001**	**+35.9**
Triglycerides (mg/dL)	0.85 (0.57–1.25)	0.65 (0.46–0.89)	**0.0001**	**+23.5**
Alkaline phosphatase (U/L)	61.9 (50.9–77.6)	65 (52.8–80.7)	0.099	
Alanine aminotransferase (U/L)	19.8 (14.8–30.1)	15.8 (11.9–21.7)	**0.0001**	**+20.2**
Aspartate aminotransferase (U/L)	18.7 (14.4–22.5)	19.3 (15.9–24.2)	0.072	
GGT (U/L)	18.9 (11.2–29.6)	22.5 (15.6–33.7)	**0.0001**	**−16.0**
Glucose (mM)	4.77 (3.10–5.26)	3.36 (2.85–4.67)	**0.0001**	**+29.6**
CRP (mg/L)	0.62 (0.23–1.55)	0.57 (0.21–1.40)	0.140	
IL-6 (pg/mL)	0.89 (0.64–1.38)	1.0 (0.68–1.41)	0.059	
TNF-α (pg/mL)	1.16 (0.93–1.60)	0.99 (0.76–1.30)	**0.0001**	**+14.7**
IGF-1 (nM)	30.8 (24.8–35.4)	26.6 (22.1–35.1)	0.081	
IGFBP-3 (nM)	127 (115–144)	114 (95.1–128)	**0.002**	**+10.2**
IGF-1/IGFBP-3	0.228 (0.181–0.268)	0.241 (0.190–0.300)	**0.013**	**−6.2**
**Urinary metabolites**				
Creatinine (mM)	13.8 (10.3–18.6)	12.3 (7.57–18.9)	**0.043**	**+10.9**
DMA (µM/mM)	26.3 (23.55–29.8)	25.4 (22.7–29.2)	**0.017**	**+3.4**
ADMA (µM/mM)	3.28 (2.75–3.88)	3.34 (2.91–3.98)	0.235	
SDMA (µM/mM)	3.59 (3.09–4.22)	3.48 (2.97–3.98)	**0.029**	**+3.1**
DMA+ADMA+SDMA (µM/mM)	33.2 (29.8–37.72)	32.6 (28.9–44.4)	**0.050**	**+1.8**
DMA+ADMA (µM/mM)	29.6 (26.5–33.7)	29.0 (26.7–33.1)	**0.042**	**+2.0**
(DMA+ADMA)/SDMA	8.35 (7.56–9.22)	8.51 (7.57–9.27)	0.363	
ADMA/SDMA	0.91 (0.80–1.08)	0.99 (0.86–1.12)	**0.0001**	**−8.1**

Data are presented as mean ± SD, percentage or median [IQR], and mean percentage difference Δ. Statistical analysis was performed using Student’s *t* test or Mann–Whitney U test as appropriate. Bold indicates statistical significance (*p* < 0.05).

**Table 3:** Comparison of white boys with white men, and of black boys with black men of the ASOS and African-PREDICT studies with respect to urinary creatinine and creatinine-corrected urinary indices of whole-body L-arginine dimethylation.

Parameter	ASOS (boys)		African-PREDICT (men)		*p*-Value	*p*-Value
	White (*n* = 41)	Black (*n* = 39)	White (*n* = 281)	Black (*n* = 292)	White vs. white	Black vs. black
Creatinine (mM)	15.9 (12.8–18.7)	15.3 (10.1–21.4)	13.8 (10.3–18.6)	12.3 (7.57–18.9)	0.053	0.123
DMA (µM/mM)	38.4 (33.846.8)	33.9 (29.0–38.8)	26.3 (23.6–29.8)	25.4 (22.7–29.2)	**0.0001**	**0.0001**
ADMA (µM/mM)	5.51 (4.91–6.30)	5.17 (4.81–6.52)	3.28 (2.75–3.88)	3.34 (2.91–3.98)	**0.0001**	**0.0001**
SDMA (µM/mM)	4.56 (4.17–5.52)	4.52 (4.06–5.07)	3.59 (3.09–4.22)	3.48 (2.97–3.98)	**0.0001**	**0.0001**
DMA+ADMA+SDMA (µM/mM)	48.5 (43.1–58.5)	44.8 (39.3–49.9)	33.2 (29.8–37.7)	32.6 (28.9–44.4)	**0.0001**	**0.0001**
DMA+ADMA (µM/mM)	44 (39.0–52.7)	39.8 (35.2–45.3)	29.6 (26.5–33.7)	29.0 (26.7–33.1)	**0.0001**	**0.0001**
(DMA+ADMA)/SDMA	8.97 (7.77–10.1)	8.66 (7.70–9.87)	8.35 (7.56–9.22)	8.51 (7.57–9.27)	**0.043**	0.259
ADMA/SDMA	1.14 (1.00–1.32)	1.21 (1.10–1.44)	0.91 (0.80–1.08)	0.99 (0.86–1.12)	**0.0001**	**0.0001**

Data are presented as median [IQR]. Statistical analysis was performed via Mann–Whitney U test. Bold indicates statistical significance.

**Table 4:** Anthropometric characteristics, urinary creatinine and creatinine-corrected indices of L-arginine dimethylation in the women of the African-PREDICT study.

Parameter	White (*n* = 312)	Black (*n* = 309)	*p* Value	Mean Δ(white−black) (%)
Characteristics				
Age (years)	24 (22–27)	25 (22–27)	**0.596**	
BMI (kg/m^2^)	23.2 (21.0–26.5)	26.0 (22.4–30.3)	**<0.0001**	**−10.8**
**Urinary metabolites**				
Creatinine (mM)	14.8 (11.1–20.0)	11.0 (6.87–17.0)	0.185	
DMA (µM/mM)	29.9 (26.2–34.5)	27.7 (24.8–32.4)	**0.001**	**+7.4**
ADMA (µM/mM)	4.09 (3.38–4.92)	4.25 (3.47–4.97)	0.594	
SDMA (µM/mM)	4.26 (3.71–4.81)	3.97 (3.40–4.57)	**0.001**	**+6.8**
DMA+ADMA+SDMA (µM/mM)	38.7 (33.9–44.4)	36.4 (32.5–41.7)	**<0.0001**	**+5.9**
(DMA+ADMA)/SDMA	8.10 (7.33–9.06)	8.25 (7.67–9.07)	0.422	
SDMA/ADMA	1.04 (0.88–1.23)	0.93 (0.81–1.08)	< 0.0001	**+10.6**

Data are presented as median [IQR]. Statistical analysis was performed via Mann–Whitney U test. Bold indicates statistical significance (*p* < 0.05).

## Supplementary Materials

The following are available online at https://www.mdpi.com/2077-0383/9/3/844/s1, Figure S1: Simplified schematic of monomethylation of the guanidine (*N*^G^) amine group of L-arginine residues in proteins, subsequent asymmetric and symmetric *N*^G^-dimethylation of the monomethylated arginine residues in proteins, their proteolysis to free ADMA and SDMA, metabolism of ADMA by DDAH to DMA, and their excretion in the urine. Proposal of the Protein-Arginine Dimethylation IndeX (PADiMeX), Table S1: Spearman correlation coefficients and P values obtained from correlations between concentrations of urinary metabolites and electrolytes in the ASOS study. Only statistically significant correlations are listed, Table S2: Spearman correlation coefficients (r) and statistical significance (P value) obtained from correlations between concentrations of urinary metabolites and electrolytes in the African-PREDICT study. Only statistically significant correlations are listed, Table S3: Comparison of white and black men and women of the African-PREDICT study with respect to urinary creatinine (mM) and creatinine-corrected (µM/mM) urinary indices of whole-body L-arginine dimethylation.
