# Supplementary material for: An Ethnic Comparison of Arginine Dimethylation and Cardiometabolic Factors in Healthy Black and White Youth: The ASOS and African-PREDICT Studies
Source: J Clin Med. 2020 Mar 20;9(3):844. doi: 10.3390/jcm9030844 (PMC7141317; doi:10.3390/jcm9030844)
Supplement: Supplementary file 1 [file jcm-09-00844-s001.pdf]

Supplement to:

An ethnic comparison of arginine-dimethylation and cardio-metabolic factors in healthy black and white youth: The ASOS and African-PREDICT studies

Alexander Bollenbach,[1] Aletta E. Schutte,[2] Ruan Kruger,[2] Dimitrios Tsikas[1,*]

[1] *Institute of Toxicology, Core Unit Proteomics, Hannover Medical School, Hannover, Germany*

[2] *Hypertension in Africa Research Team (HART), MRC Research Unit for Hypertension and Cardiovascular Disease, North-West University, Potchefstroom, South Africa*

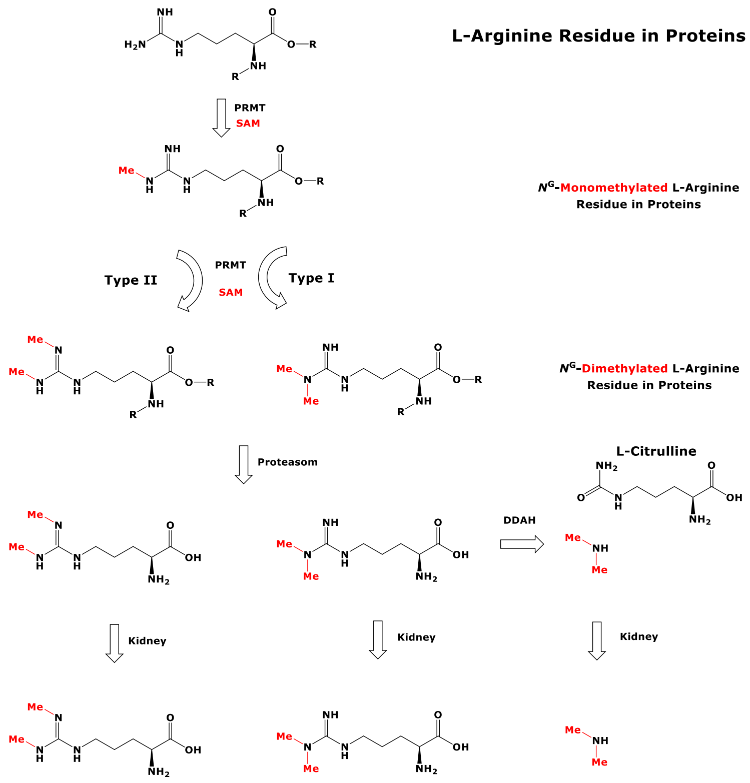

SDMA | ADMA | DMA

Symmetric Arg Methylation

sPADIMEX = SDMA

Asymmetric Arg Methylation

aPADIMEX = ADMA + DMA

toPADIMEX = ADMA + DMA + SDMA

a/sPADIMEX = (ADMA + DMA) / SDMA

Figure S1. Simplified schematic of monomethylation of the guanidine (N^G) amine group of L-arginine residues in proteins, subsequent asymmetric and symmetric N^G-dimethylation of the monomethylated arginine residues in proteins, their proteolysis to free ADMA and SDMA, metabolism of ADMA by DDAH to DMA, and their excretion in the urine. Proposal of the **P**rotein-**A**rginine **Di**methylation Inde**X** (PADiMeX).

Abbreviations: ADMA, asymmetric dimethylarginine; DMA, dimethylamine; SDMA, symmetric dimethylarginine; PRMT, protein-arginine methyltransferase; DDAH, dimethylarginine dimethylaminohydrolase; SMA, *S*-Adenosyl-methionine

Table S1 Spearman correlation coefficients and *P* values obtained from correlations between concentrations of urinary metabolites and electrolytes in the ASOS study. Only statistically significant correlations are listed.

	Blacks	Whites	ALL	Blacks	Whites	ALL	Blacks	Whites	ALL	Blacks	Whites	ALL
	**DMA**			**ADMA**			**SDMA**			**DMA+ADMA+SDMA**		
Creatinine	R=0.936<br>p=0.0001	R=0.657<br>p=0.0001	R=0.821<br>p=0.0001	R=0.909<br>p=0.0001	r=0.820<br>p=0.0001	r=0.876<br>p=0.0001	r=0.950<br>p=0.0001	r=0.817<br>p=0.0001	r=0.903<br>p=0.0001	r=0.950<br>p=0.0001	r=0.702<br>p=0.0001	r=0.853<br>p=0.0001
DMA	X	X	X	r=0.891<br>p=0.0001	r=0.752<br>p=0.0001	r=0.823<br>p=0.0001	r=0.941<br>p=0.0001	r=0.744<br>p=0.0001	r=0.855<br>p=0.0001	R=0.995<br>p=0.0001	R=0.992<br>p=0.0001	R=0.994<br>p=0.0001
ADMA	R=0.891<br>p=0.0001	r=0.752<br>p=0.0001	R=0.823<br>p=0.0001	X	X	X	R=0.894<br>p=0.0001	R=0.841<br>p=0.0001	R=0.869<br>p=0.0001	R=0.916<br>p=0.0001	R=0.795<br>p=0.0001	R=0.860<br>p=0.0001
SDMA	R=0.941<br>p=0.0001	R=0.744<br>p=0.0001	R=0.855<br>p=0.0001	R=0.894<br>p=0.0001	R=0.841<br>p=0.0001	R=0.869<br>p=0.0001	X	X	X	R=0.957<br>p=0.0001	R=0.794<br>p=0.0001	R=0.891<br>p=0.0001
DMA+ADMA+<br>SDMA	R=0.995<br>p=0.0001	R=0.992<br>p=0.0001	R=0.994<br>p=0.0001	R=0.916<br>p=0.0001	R=0.795<br>p=0.0001	R=0.860<br>p=0.0001	R=0.957<br>p=0.0001	R=0.794<br>p=0.0001	R=0.891<br>p=0.0001	X	X	X
ADMA/SDMA	R=-0.374<br>p=0.019		R=-0.340<br>p=0.002				R=-0.479<br>p=0.002	R=-0.620<br>p=0.0001	R=-0.551<br>p=0.0001	R=-0.367<br>p=0.022	R=-0.317<br>p=0.044	R=-0.359<br>p=0.0001
(DMA+ADMA)/<br>SDMA	R=0.330<br>p=0.0399	R=0.503<br>p=0.0008	R=0.418<br>p=0.0001								R=0.439<br>p=0.004	R=0.356<br>p=0.001
Chloride	R=0.462<br>p=0.003		R=0.252<br>p=0.025	R=0.442<br>p=0.0048		R=0.269<br>p=0.0164	R=0.546<br>p=0.0003		R=0.333<br>p=0.0027	R=0.455<br>p=0.004		R=0.264<br>p=0.019
Potassium	R=0.518<br>p=0.00073		R=0.344<br>p=0.0019	R=0.484<br>p=0.0017		R=0.256<br>p=0.022	R=0.523<br>p=0.0006		R=0.355<br>p=0.0013	R=0.498<br>p=0.001		R=0.343<br>p=0.002
Sodium	R=0.370<br>p=0.020		R=0.223<br>p=0.0477	R=0.321<br>p=0.045			R=0.463<br>p=0.002		R=0.297<br>p=0.0078	R=0.365<br>p=0.022		R=0.232<br>p=0.039

Table S2 Spearman correlation coefficients (*r*) and statistical significance (*P* value) obtained from correlations between concentrations of urinary metabolites and electrolytes in the African-PREDICT study. Only statistically significant correlations are listed.

	Blacks	Whites	ALL	Blacks	Whites	ALL	Blacks	Whites	ALL	Blacks	Whites	ALL
	**DMA**			**ADMA**			**SDMA**			**DMA+ADMA+SDMA**		
Creatinine	R=0.943 p=0.0001	R=0.895 p=0.001	R=0.925 p=0.0001	R=0.935 p=0.0001	R=0.867 p=0.0001	R=0.908 p=0.0001	R=0.908 p=0.0001	R=0.861 p=0.0001	R=0.892 p=0.0001	R=0.948 p=0.0001	R=0.901 p=0.0001	R=0.931 p=0.0001
DMA	X	X	X	R=0.945 p=0.0001	R=0.877 p=0.0001	R=0.916 p=0.0001	R=0.929 p=0.0001	R=0.879 p=0.0001	R=0.911 p=0.0001	R=0.998 p=0.0001	R=0.996 p=0.0001	R=0.998 p=0.0001
ADMA	R=0.945 p=0.0001	R=0.877 p=0.0001	R=0.916 p=0.0001	X	X	X	R=0.919 p=0.0001	R=0.866 p=0.0001	R=0.900 p=0.0001	R=0.959 p=0.0001	R=0.903 p=0.0001	R=0.935 p=0.0001
SDMA	R=0.929 p=0.0001	R=0.879 p=0.0001	R=0.911 p=0.0001	R=0.919 p=0.0001	R=0.866 p=0.0001	R=0.900 p=0.0001	X	X	X	R=0.946 p=0.0001	R=0.906 p=0.0001	R=0.931 p=0.0001
DMA+ADMA+ SDMA	R=0.998 p=0.0001	R=0.996 p=0.0001	R=0.998 p=0.0001	R=0.959 p=0.0001	R=0.903 p=0.0001	R=0.935 p=0.0001	R=0.946 p=0.0001	R=0.906 p=0.0001	R=0.931 p=0.0001	X	X	X
SDMA/ADMA				R=-0.122 p=0.037	R=-0.341 p=0.0001	R=-0.213 p=0.0001	R=0.204 p=0.0005		R=0.159 p=0.0001			
(DMA+ADMA)/ SDMA							R=-0.251 p=0.0001	R=-0.306 p=0.0001	R=-0.277 p=0.0001			
Body height	R=0.127 p=0.030		R=0.115 p=0.006	R=0.153 p=0.005		R=0.102 p=0.017	R=0.153 p=0.009		R=0.102 p=0.015	R=0.135 p=0.021		R=0.116 p=0.006
Body weight	R=0.202 p=0.001		R=0.136 p=0.001	R=0.182 p=0.0004		R=0.112 p=0.011	R=0.182 p=0.002		R=0.112 p=0.007	R=0.202 p=0.001		R=0.133 p=0.001
BMI	R=0.148 p=0.011		R=0.114 p=0.006	R=0.139 p=0.017		R=0.082 p=0.049	R=0.117 p=0.047		R=0.091 p=0.029	R=0.145 p=0.013		R=0.111 p=0.008
Waist circumference	R=0.151 p=0.010		R=0.090 p=0.0031	R=0.139 p=0.006		R=0.102 p=0.014	R=0.139 p=0.017		R=0.102 p=0.015	R=0.152 p=0.009		R=0.087 p=0.038
Neck circumference	R=0.132 p=0.024		R=0.103 p=0.014						R=0.084 p=0.045	R=0.126 p=0.031		R=0.097 p=0.020
Hip circumference	R=0.133 p=0.023		R=0.116 p=0.005	R=0.103 p=0.035		R=0.081 p=0.041				R=0.130 p=0.027		R=0.113 p=0.007
Waist-hip ratio									R=0.083 p=0.046			

Blood glucose	R=0.215<br>p=0.0002		R=0.113<br>p=0.007	R=0.190<br>p=0.001	R=-0.198<br>p=0.001		R=0.198<br>p=0.001			R=0.213<br>p=0.002		R=0.100<br>p=0.017
IL-6	R=0.143<br>p=0.015			R=0.147<br>p=0.002	R=0.080<br>p=0.001	R=0.103<br>p=0.014			R=0.123<br>p=0.003	R=0.146<br>p=0.012		R=0.114<br>p=0.006
IGFBP-3	R=-0.200<br>p=0.0087			R=-0.167<br>p=0.029			R=-0.161<br>p=0.035			R=-0.191<br>p=0.012		
GGT					R=-0.073<br>p=0.027							
HDL					R=-0.112<br>p=0.0002	R=-0.089<br>p=0.0004						
LDL					R=-0.062<br>p=0.017							
IGF-1/IGFBP-3						R=0.129<br>p=0.018			R=0.114<br>p=0.037			

Table S3 Comparison of white and black men and women of the African-PREDICT study with respect to urinary creatinine (mM) and creatinine-corrected (µM/mM) urinary indices of whole-body L-arginine dimethylation.

Group	A	B	C	D	*P* value	*P* value	*P* value	*P* value	*P* value	*P* value	*P* value
Gender, Ethnicity	Male white (*n*=281)	Female white (*n*=312)	Male black (*n*=292)	Female black (*n*=309)	ANOVA	A vs. B	A vs. C	A vs. D	B vs. C	B vs. D	C vs. D
Age (years)	25 [22-27]	24 [22-27]	24 [22-27]	25 [22-27]	0.379						
BMI (kg/m²)	26.2 [23.4-29.2]	23.2 [21-26.46]	21.4 [19.4-24.2]	26 [22.4-30.3]	**<0.0001**	**<0.0001**	**<0.0001**	0.945	**<0.0001**	**<0.0001**	**<0.0001**
Creatinine (mM)	13.8 [10.3-18.6]	14.8 [11.1-20]	12.3 [7.57-18.9]	11 [6.87-17]	**0.002**	**0.024**	0.413	**0.002**	0.573	0.881	0.185
DMA (µM/mM)	26.3 [23.6-29.8]	29.9 [26.2-34.5]	25.4 [22.7-29.2]	27.7 [24.8-32.4]	**<0.0001**	**<0.0001**	**0.013**	0.975	**<0.0001**	**0.001**	**0.002**
ADMA (µM/mM)	3.28 [2.75-3.88]	4.09 [3.38-4.92]	3.34 [2.91-3.98]	4.25 [3.47-4.97]	**<0.0001**	**<0.0001**	0.929	**<0.0001**	**<0.0001**	0.594	**<0.0001**
SDMA (µM/mM)	3.59 [3.09-4.22]	4.26 [3.71-4.81]	3.48 [2.97-3.98]	3.97 [3.4-4.57]	**<0.0001**	**<0.0001**	0.990	**0.022**	**<0.0001**	**0.001**	**0.007**
DMA+ADMA+SDMA (µM/mM)	33.2 [29.8-37.7]	38.7 [33.9-44.4]	32.6 [28.9-37]	36.4 [32.5-41.7]	**<0.0001**	**<0.0001**	**0.027**	0.336	**<0.0001**	**<0.0001**	**<0.0001**
SDMA/ADMA	1.1 [0.94-1.26]	1.04 [0.88-1.23]	1.01 [0.9-1.16]	0.93 [0.81-1.08]	**<0.0001**	0.916	0.892	**0.005**	0.507	**<0.0001**	**0.043**
(DMA+ADMA)/SDMA	8.35 [7.56-9.22]	8.1 [7.33-9.06]	8.51 [7.57-9.27]	8.25 [7.67-9.07]	**0.027**	0.628	0.071	**0.037**	0.568	0.422	0.997

Data are represented as median with interquartile range. Statistical analysis was perfomed using One-way ANOVA with Tukey's correction. Bold indicates statistical significance ($p < 0.05$).
